# Protection Versus Pathology in Aviremic and High Viral Load HIV-2 Infection—The Pivotal Role of Immune Activation and T-cell Kinetics

**DOI:** 10.1093/infdis/jiu165

**Published:** 2014-05-05

**Authors:** Andrea Hegedus, Samuel Nyamweya, Yan Zhang, Sheila Govind, Richard Aspinall, Alla Mashanova, Vincent A. A. Jansen, Hilton Whittle, Assan Jaye, Katie L. Flanagan, Derek C. Macallan

**Affiliations:** 1 Infection and Immunity Research Institute, St George's, University of London, United Kingdom; 2 Medical Research Council (UK), The Gambia, West Africa; 3 Translational Medicine Group, Cranfield Health, Cranfield University; 4 School of Biological Sciences, Royal HollowayUniversity of London, United Kingdom

**Keywords:** CD4, CD8, HIV-2, HIV-1, HIV pathogenesis, immune activation, immune memory, T-cell

## Abstract

***Background.*** Many human immunodeficiency virus (HIV)–2-infected individuals remain aviremic and behave as long-term non-progressors but some progress to AIDS. We hypothesized that immune activation and T-cell turnover would be critical determinants of non-progressor/progressor status.

***Methods.*** We studied 37 subjects in The Gambia, West Africa: 10 HIV-negative controls, 10 HIV-2-infected subjects with low viral loads (HIV-2-LV), 7 HIV-2-infected subjects with high viral loads (HIV-2-HV), and 10 with HIV-1 infection. We measured in vivo T-cell turnover using deuterium-glucose labeling, and correlated results with T-cell phenotype (by flow cytometry) and T-cell receptor excision circle (TREC) abundance.

***Results.*** Immune activation (HLA-DR/CD38 coexpression) differed between groups with a significant trend: controls <HIV-2-LV <HIV-1 <HIV-2-HV (*P* < .01 for all cell types). A similar trend was observed in the pattern of in vivo turnover of memory CD4^+^ and CD8^+^ T-cells and TREC depletion in naive CD4^+^ T-cells, although naive T-cell turnover was relatively unaffected by either infection. T-cell turnover, immune activation, and progressor status were closely associated.

***Conclusions.*** HIV-2 non-progressors have low rates of T-cell turnover (both CD4^+^ and CD8^+^) and minimal immune activation; high viral load HIV-2 progressors had high values, similar to or exceeding those in HIV-1 infection.

Human immunodeficiency virus (HIV)–2 infection represents a natural model for retroviral disease in which non-progression is the norm. Although HIV-2 can cause an immunodeficiency syndrome indistinguishable from HIV-1-induced AIDS [1, 2], many HIV-2-infected individuals do not develop immunodeficiency within their lifetime and retain stable CD4 lymphocyte counts for many years [3]. Indeed in some West African populations, aviremic HIV-2 infection has no independent impact on survival [4]. The marker of this low-risk state is a low or undetectable plasma viral load (VL) [3, 5], which remains stable for many years [4]. Aviremic or low viral load HIV-2 infection thus mirrors the therapeutic target state of “functional cure,” characterized by “long-term control of HIV in the absence of antiretroviral treatment” (ART) [6] and represents an instructive model for mitigation of retroviral pathology [1, 2].

This propensity for non-progression cannot be explained solely in virological terms. First, in vitro cytopathogenicity for CD4^+^ T-cells is similar for HIV-1 and HIV-2 [7]. Second, although high HIV-2 viral loads indicate patients likely to progress [3, 8] and correlate with mortality, as for HIV-1 [9], advanced pathogenic HIV-2 occurs at relatively low viral loads [10], approximately one log_10_ lower than those in HIV-1-infected groups with similar mortality [9]. It seems likely, therefore, that HIV-2 non-progression results from a more propitious immune response comprising (1) better protection, and (2) less immunopathology.

Several lines of evidence support the concept of greater immune protection. First, in HIV-2 infection, CD4^+^ T-cells retain better proliferative capacity, remain less differentiated, and elicit more polyfunctional responses than in HIV-1 infection [11]. Second, CD8^+^ T-cell responses, while retaining functional flexibility [12], appear highly focused [13] with abundant polyfunctional, relatively undifferentiated, Gag-specific effectors [14]. Furthermore, HIV-1/HIV-2 coinfection appears to protect against HIV-1 pathogenicity [15], although this observation is contentious [16] and not supported by meta-analysis [17].

Concurrently, immune responses in HIV-2 infection appear less immunopathogenic. In HIV-1 infection, immune activation is a pivotal mediator of immunopathology, strongly predicting disease progression [18]. HIV-2 cohorts have lower levels of immune activation, intermediate between seronegative controls and HIV-1-infected groups [19], consistent with the “less activation/better outcome” paradigm. However, such cohorts typically include few HIV-2 subjects with detectable/high VL and low CD4 counts. When analyzed separately, HIV-2 high viral load progressors demonstrate highly activated immune profiles, similar to those in HIV-1 infection [20]; indeed, HIV-2 may induce greater immune activation per unit viremia [21]. In HIV-2, immune activation correlates with clinical parameters, such as wasting, functional performance, and mortality [20], and soluble markers of immune activation (β2-microglobulin, neopterin, and sUPAR) predict mortality in the same way for HIV-2 and HIV-1, despite their widely disparate viral loads and CD4 counts [21]. Excessive immune activation in the subpopulation of HIV-2 patients with viremia may explain why they develop AIDS or die at relatively high CD4 counts [19]. Once profound CD4 lymphopenia (<200 cells/μL) has developed, mortality is similar for both HIV-2 and HIV-1 infection [20].

The well-recognized link between immune activation and immunopathology remains poorly explained, but exhaustion of homeostatic T-cell replacement by accelerated turnover, a characteristic feature of HIV-1 infection [22–24], may be critical. Memory T-cells appear most affected, irrespective of viral tropism [25]; by contrast, in early disease, naive T-cells remain relatively unaffected [25]. In later-stage disease, rates of loss of naive cells, whether by direct viral targeting, activation, or accelerated homeostatic turnover, may exceed their normally slow homeostatic replacement [26, 27]. We therefore hypothesized that long-term survivor, low viral load HIV-2-infected subjects would have low levels of immune activation and low (normal) rates of T-cell turnover, while HIV-2-infected subjects with high viral loads would have accelerated turnover, similar to those in HIV-1-infected individuals. To test these hypotheses, we measured in vivo T-cell turnover using oral deuterium-glucose labeling [28] and compared the results to activation status by immunophenotyping, and replicative history from T-cell receptor excision circle (TREC) abundance, in asymptomatic HIV-1- and HIV-2-infected subjects in an HIV-2-endemic area of Africa.

## METHODS

### Ethics Statement

All subjects gave written informed consent; the study was approved by the Gambia Government/Medical Research Council Joint Ethics Committee. All procedures were conducted in accordance with the ethical standards of the Helsinki Declaration.

### Clinical

Four groups of subjects were recruited from a clinical cohort at the Medical Research Council (UK) in Fajara, The Gambia, West Africa: (1) healthy HIV-negative controls, (2) HIV-2-infected subjects with low plasma viral loads (<100 viral copies/mL), (3) HIV-2-infected subjects with high viral loads (>1000 viral copies/mL), thus considered likely to progress rapidly to AIDS, and, (4) HIV-1-infected subjects. A CD4 count of ≥500 cells/μL at screening was an entry criterion, as we wished to investigate early events in HIV pathogenesis. All were ART-naive, aged ≥18 years, clinically well with no apparent intercurrent illness, no fever, a negative malaria film, a hemoglobin level of ≥10 g/dL, and fasting glucose ≤6 mmol/L. Target recruitment was 10 per group; however, as few HIV-2-infected patients have detectable viral loads, recruitment to this group only reached 7 subjects. Thirty-eight subjects entered the study but 1 control subject declined follow-up.

### Immunophenotyping and T-cell Receptor Excision Circle Abundance Analysis

Peripheral blood mononuclear cells were stained for expression of CD3, CD4, CD8, CD45R0, CD45RA, human leukocyte antigen (HLA)–DR, CD38, Ki67, CD95, Annexin V, and programmed death-1 (PD-1) in 3 overlapping panels (see Supplementary Information). Data acquired by multicolor flow cytometry (CyAn, Beckman Coulter) were analyzed by FlowJo software (Tree Star) (Supplementary Figure 1). T-cell receptor excision circle abundance (TREC) quantification was performed as previously described [29] (Supplementary Information) and expressed as TREC per 10^5^ CD4^+^ or CD8^+^ CD45R0^−^ T-cells.

### In Vivo Turnover Measurements

Proliferation and disappearance rates of lymphocyte subpopulations were measured using deuterium-glucose labeling [28, 30]. Subjects received 60g of 6,6–^2^H_2_-glucose (Cambridge Isotopes) orally in half-hourly aliquots over 10 hours, as described [31], monitoring plasma glucose deuterium-enrichment for precursor estimation. Blood was taken 4, 10, and 21 days post-labeling and peripheral blood mononuclear cell fractions sorted by negative-selection for CD45R0 and CD45RA into “naive” (CD45R0^−^) and “memory” (CD45RA^−^) subpopulations, then by positive-selection for CD8 and CD4 using antibody-coated magnetic beads (Miltenyi Biotec) (Supplementary Information) [31]. Purities were confirmed with both CD45RA and CD45R0. (Definition of “naive” cells by CD45R0^−^ would have an expected specificity of 97%–98% for CD4 cells and 87%–93% for CD8 cells [32].) Lymphocyte subpopulations were analyzed for DNA deuterium-labeling by gas chromatography mass spectrometry, as described [28, 33].

### Modeling/Statistics

In order to compare in vivo T-cell proliferation rates, we modeled the incorporation of deuterium into deoxyadenosine of cellular DNA, as previously described (Supplementary Information) [34], to derive an in vivo proliferation rate, *p*, which relates to the whole population, and a disappearance/death rate, d*, which relates only to recently divided, labeled cells. Modeling was performed using Sigmaplot (Systat Software) and statistical comparisons using Prism (GraphPad Software). Comparisons were nonparametric (Kruskal–Wallis), except where data passed D'Agostino–Pearson normality testing, when parametric analysis of variance was used.

## RESULTS

### Clinical Details

Clinical details are summarized in Table 1. As expected from the entry criteria (CD4 >500 cells/μL), CD4 counts were similar between groups, while CD8 counts were higher in HIV-1-infected subjects than in other groups. HIV-1-infected subjects tended to have a lower body weight, while controls were younger than HIV-infected subjects in the HIV-1 and HIV-2-HV groups. Notably, low viral load HIV-2 subjects had documented non-progression for a median of 11.8 years.

**Table 1.** Subject Characteristics

	Controls	HIV-2-LV	HIV-2-HV	HIV-1	*P* Value^a^
N	10	10	7	10	
Gender (F:M)	7:3	8:2	7:0	6:4	
Age (years)	26.5 (23–35)	41.0 (38–46)	38.0 (35–52)	42.0 (29–55)	<.005
Viral load (copies/mL)	N/A	<100	2834 (2028–121 199)	186 347 (53 249–486 378)	<.001
CD4 count (cells/µL)	872 (747–1286)	946 (681–1019)	761 (530–980)	780 (575–854)	.263
CD4%	40.5 (38–45)	35.5 (32–45)	37.0 (32–39)	27.5 (35–21)	.004
CD8 count (cells/µL)	689 (456–774)	750 (549–1047)	637 (427–804)	1076 (926–1600)	.010
Weight (kg)	63.7 (61.4–84.1)	71.8 (62.3–80.7)	66.4 (58.7–96.2)	54.8 (50.0–63.2)	.023
Time in cohort (years)	N/A	11.8 (4.1–14.8)	5.7 (1.0–11.7)	1.3 (0.9–5.5)	.020

Values are median (IQR).

Abbreviations: ANOVA, analysis of variance; HIV, human immunodeficiency virus; HIV-2-HV, HIV-2 infected with high viral load (>1000 copies/mL); HIV-2-LV, HIV-2 infected with low viral load (<100 copies/mL); IQR, interquartile range; N/A, not applicable.

^a^ *P* represents comparison of groups by ANOVA (Kruskal–Wallis).

### HIV-2 Non-progressors are Characterized by Low Levels of Immune Activation in Naive and Memory T-cell Populations

**Figure 1.**
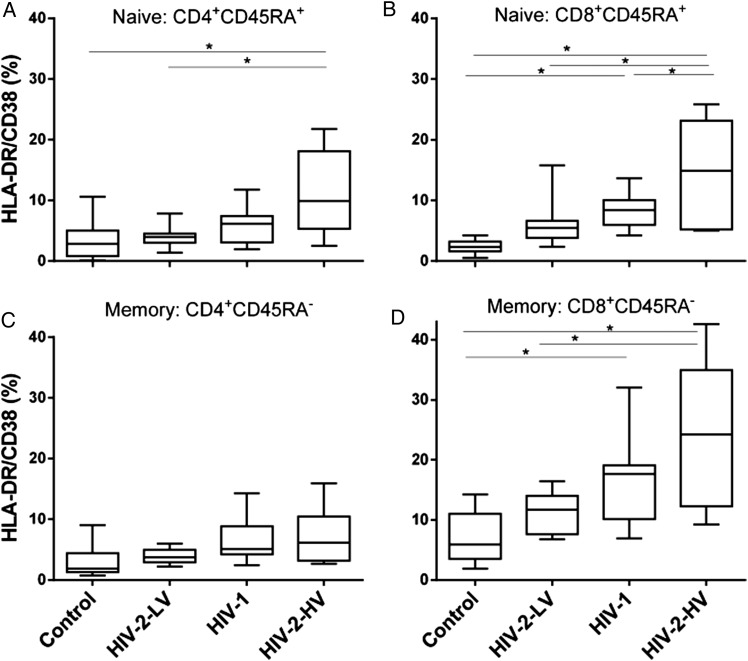
HIV-1 and HIV-2 at high viral loads induce immune activation in both naive and memory T-cell subsets. Values represent proportion of T-cells coexpressing HLA-DR and CD38, as median, interquartile (box) and range (whiskers) within the following populations: (*A*) CD4^+^CD45RA^+^ and (*B*) CD8^+^CD45RA^+^ “naive” cells; (*C*) CD4^+^CD45RA^−^ and (*D*) CD8^+^CD45RA^−^ “memory” cells. HIV-2-LV and HIV-2-HV refer to low and high viral load HIV-2-infected subjects. Group differences tested by ANOVA: (*A*) 0.012, (*B*) <0.001, (*C*) 0.059, (*D*) <0.001; *P < .05 by post hoc (Tukey) test. Hierarchical trend analysis across groups ranked in this order, *P* < .01 for all cell types. No differences were observed between memory and naive CD4^+^ T-cells, median values, all subjects, CD45RA^+^ 4.51% versus CD45RA^−^ 4.30% (*P* = .554), but in CD8^+^ cells, activation was greater in memory cells, median CD45RA^+^: 5.72 versus CD45RA^−^: 12.8% (*P* < .0001, Wilcoxon matched-pairs signed rank test). Abbreviations: ANOVA, analysis of variance; HIV, human immunodeficiency virus; HIV-2-HV, HIV-2 infected with high viral load (>1000 copies/mL); HIV-2-LV, HIV-2 infected with low viral load (<100 copies/mL); HLA, human leukocyte antigen.

When we investigated levels of immune activation (HLA-DR/CD38 coexpression) by flow cytometry within CD4^+^- and CD8^+^-naive and memory T-cell subsets (defined by CD45RA^+^ or CD45R0^+^ expression, respectively), we found a similar pattern for all 4 T-cell subpopulations. Immune activation levels remained low in HIV-2 low viral load subjects, close to control values, but were highest in HIV-2 subjects with high viral loads (Figure 1), similar to or exceeding levels in HIV-1-infected subjects (Table 2). Although this has previously been observed in other cohorts [20], a novel aspect here is the separation of naive and memory cells, cognizant that involvement of the naive pool may be pivotal in disease progression. We found that activation was induced in both naive and memory compartments in HIV-1 and HIV-2-HV, but not significantly in either compartment in HIV-2-LV. In all T-cell subgroups, median immune activation followed the same trend: controls <HIV-2-LV <HIV-1 <HIV-2-HV. When ranked in this order using a categorical variable (1–4), correlation analysis demonstrated a highly significant hierarchy for all four T-cell subpopulations (Figure 1). In order to investigate whether immune activation cosegregated with other parameters according to subject group, this hierarchical ranking was used in subsequent analyses.

**Table 2.** Immune Activation, Ki67 Expression, and TREC Content in Lymphocyte Subsets in Control and HIV-Infected Subjects

	Controls	HIV-2-LV	HIV-2-HV	HIV-1	*P* Value^a^
HLA-DR/CD38 coexpression (%)					
CD4^+^CD45RA^+^	2.83 (1.45–4.69)	3.98 (3.39–4.47)	9.94 (6.40–16.0)	6.15 (3.92–6.95)	.012
CD8^+^CD45RA^+^	2.80 (2.05–3.48)	5.47 (4.15–6.05)	14.89 (6.97–21.1)	8.41 (6.13–9.69)	<.001
CD4^+^CD45RA^−^	2.33 (1.47–3.48)	3.77 (3.04–6.05)	6.14 (3.59–21.1)	5.12 (4.46–9.69)	.059
CD8^+^CD45RA^−^	5.91 (3.96–10.1)	11.69 (7.88–13.3)	24.24 (14.51–31.8)	17.67 (11.3–18.9)	<.001
Ki67 expression (%)					
CD4^+^CD45RA^+^	1.12 (0.56–2.01)	1.13 (0.95–1.69)	3.30 (1.76–9.2)	1.60 (1.25–2.11)	.082
CD8^+^CD45RA^+^	3.22 (1.88–4.06)	1.93 (1.78–2.34)	4.51 (3.30–11.0)	2.69 (2.22–3.29)	.052
CD4^+^CD45RA^−^	4.88 (3.07–4.06)	4.84 (4.14–2.34)	9.14 (7.09–11.0)	6.56 (5.01–3.29)	.055
CD8^+^CD45RA^−^	5.61 (4.19–6.90)	4.99 (3.53–6.90)	11.06 (6.93–16.1)	8.14 (7.05–11.1)	.035
sjTREC (log copies/10^5^ cells)					
CD4^+^CD45R0^−^	4.43 (4.17–4.51)	4.13 (3.63–4.17)	3.58 (3.38–3.65)	4.10 (3.33–4.41)	.004
CD8^+^CD45R0^−^	4.23 (4.06–4.40)	4.03 (3.90–4.21)	3.49 (3.33–4.02)	4.00 (3.94–4.11)	.189

Values represent median (IQR).

Abbreviations: ANOVA, analysis of variance; HIV, human immunodeficiency virus; HIV-2-HV, HIV-2 infected with high viral load (>1000 copies/mL); HIV-2-LV, HIV-2 infected with low viral load (<100 copies/mL); IQR, interquartile range; TREC, T-cell receptor excision circle.

^a^ *P* value represents comparison of groups by ANOVA.

Markers of cell death, such as the early apoptotic marker Annexin V and CD95 (Fas or APO-1), were similar between groups, although an excess in Bcl-2 expression was seen in 3 out of 4 T-cell subsets in HIV-2-HV subjects. PD-1 expression was higher on memory than naive T-cells as expected, but no significant differences were found between HIV-infected and control subjects. The primary phenotypic difference between T-cells in HIV-2 non-progressors and progressors therefore appears to relate to their very disparate levels of immune activation.

### Accelerated Turnover Occurs Predominantly in Memory T-cell Compartments in High Viral Load HIV-2 and HIV-1 Infection

T-cell turnover was assessed using three complementary approaches: Ki67 expression by flow cytometry, in vivo turnover from deuterium-labeling, and TREC content.

#### Ki67 Expression

**Figure 2.**
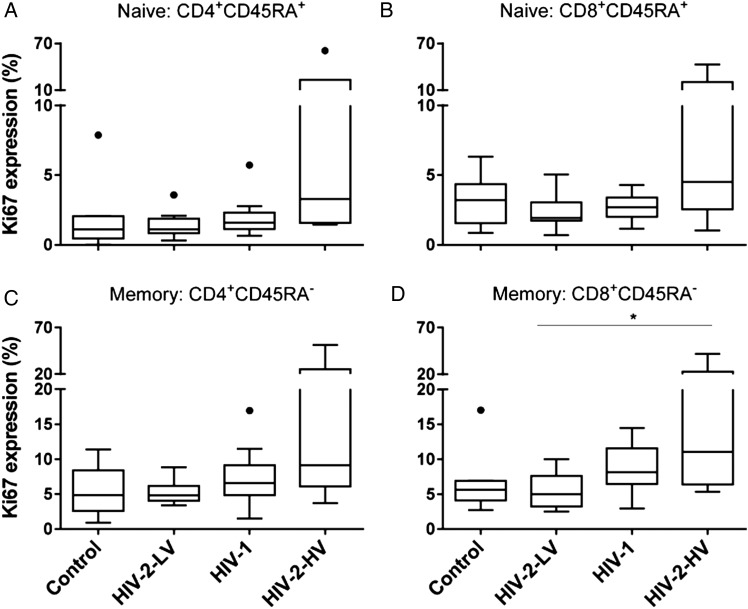
Ki67 expression in naive and memory T-cells in controls and HIV infection. Values represent proportion of cells expressing Ki67 within T-cells populations: (*A*) CD4^+^CD45RA^+^ and (*B*) CD8^+^CD45RA^+^ “naive” cells; (*C*) CD4^+^CD45RA^−^ and (*D*) CD8^+^CD45RA^−^ “memory” cells. Line, box and whiskers represent median, IQR and range; outliers shown as dots using Tukey criteria. Group differences tested by ANOVA: (*A*) 0.082, (*B*) 0.052, (*C*) 0.055, (*D*) 0.035; *P < .05 by post hoc (Tukey) test. Trend analysis, *P* < .05 for both CD4^+^ and CD8^+^ memory (CD45R0^+^) populations. Abbreviations: ANOVA, analysis of variance; HIV, human immunodeficiency virus; HIV-2-HV, HIV-2 infected with high viral load (>1000 copies/mL); HIV-2-LV, HIV-2 infected with low viral load (<100 copies/mL); IQR, interquartile range.

The nuclear protein antigen Ki67 was used as an indirect marker for “proliferation,” being present during cell cycle but not in G0 (resting) cells. As expected, Ki67 expression was generally low in naive T-cells, commensurate with their slow turnover [26, 27, 35], but tended to be elevated in naive cells from HIV-2 subjects with high viral loads (Table 2). For memory cells, Ki67 expression was highest in HIV-2-HV subjects, 9.1% and 11.1% in CD4^+^ and CD8^+^ cells, respectively, about twice control values, 4.9% and 5.6% (Table 2). By contrast, Ki67 levels in HIV-2-LV remained similar to control values. Ki67 expression followed the same hierarchy across groups (controls <HIV-2-LV <HIV-1 <HIV-2-HV; *P* < .05 for both CD4^+^ and CD8^+^; Figure 2) as immune activation.

#### In Vivo Turnover

Enrichment curves from in vivo deuterated glucose-labeling showed lower enrichment levels in naive cells than in memory cells, indicative of slower turnover, as previously described [28]. When compared between subject groups, HIV-2-HV subjects had significantly higher enrichment peaks in memory cells than control subjects (*P* < .05 for both CD4^+^ and CD8^+^; Table 3). Magnitude of peak enrichment demonstrated the same hierarchy across groups as seen with immune activation and Ki67 expression (controls <HIV-2-LV <HIV-1 <HIV-2-HV) in naive CD8^+^ cells and both CD4^+^ and CD8^+^ memory cells (Table 3).

**Table 3.** Turnover Rates of T-cell Subsets

	Controls	HIV-2-LV	HIV-2-HV	HIV-1	*P* Value
Naive T-cells (CD45R0^−^)					
CD4^+^					
Peak (%)	0.34 (0.30–0.79)	0.61 (0.41–0.79)	0.53 (0.23–0.93)	0.45 (0.25–0.85)	.520
p (%/day)	0.47 (0.34–0.56)	0.44 (0.11–0.72)	0.53 (0.20–1.13)	0.40 (0.23–0.84)	.492
T_2_ (days)	148 (124–229)	142 (92–227)	137 (69–409)	173 (83–336)	.806
CD8^+^					
Peak	0.44 (0.22–0.66)	0.47 (0.37–0.65)	0.66 (0.48–0.96)	0.88 (0.47–1.01)	.019
*p* (%/day)	0.30 (0.23–0.52)	0.38 (0.23–0.80)	0.66 (0.45–0.99)	0.69 (0.33–1.61)	.136
T_2_ (days)	232 (135–312)	182 (90–289)	105 (72–176)	100 (43–210)	.182
Memory T-cells (CD45RA^−^)					
CD4^+^					
Peak (%)	1.08 (0.77–1.70)	1.46 (1.22–1.94)	2.32 (1.12–3.57)	1.85 (1.33–3.73)	.028
*p* (%/day)	1.30 (1.00–2.47)	1.98 (1.30–2.50)	1.87 (1.57–6.72)	2.36 (1.52–5.19)	.047
T_2_ (days)	54 (28–72)	35 (28–53)	37 (10–44)	29 (13–46)	.055
CD8^+^					
Peak (%)	0.85 (0.58–1.47)	1.50 (0.97–1.82)	2.65 (1.47–5.02)	1.60 (1.00–3.34)	.008
*p* (%/day)	1.55 (0.71–2.06)	1.85 (1.20–2.29)	3.44 (2.44–7.11)	2.24 (1.10–5.27)	.010
T_2_ (days)	45 (35–98)	38 (30–58)	20 (9–28)	32 (13–63)	.031

Peak is the maximum level of isotope labeling, corrected for glucose enrichment to derive an equivalent for 1 day's labeling; *p*, modeled proliferation rate constant; T_2_, doubling time of cells, within that pool, calculated as ln(2)/*p*. Values are median (IQR). *P* values by ANOVA; for T_2_ data was log-transformed.

Abbreviations: ANOVA, analysis of variance; HIV, human immunodeficiency virus; HIV-2-HV, HIV-2 infected with high viral load (>1000 copies/mL); HIV-2-LV, HIV-2 infected with low viral load (<100 copies/mL); IQR, interquartile range.

**Figure 3.**
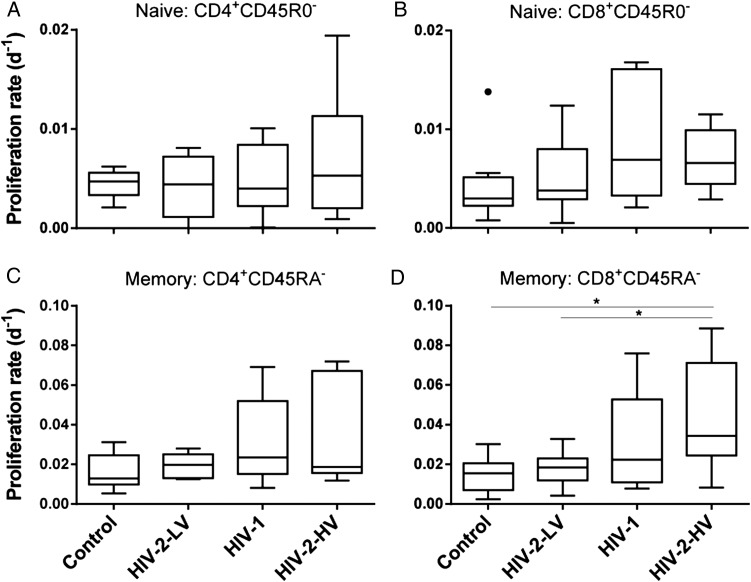
Proliferation rates of T-cell subpopulations in controls and HIV infection. Modeled proliferation rate constants (*p*) in CD4^+^ and CD8^+^ T-cell subpopulations for (*A*) CD4^+^CD45 R0^−^ and (*B*) CD8^+^CD45 R0^−^ “naive” cells; and (*C*) CD4^+^CD45RA^−^ and (*D*) CD8^+^CD45R0A^−^ “memory” cells. Line, box, and whiskers represent median, IQR, and range, respectively; outliers shown as dots using Tukey criteria. Note different scales for naive and memory cells. There was a significant trend across groups for CD4^+^ and CD8^+^ memory cells (*P* < .006, .001, respectively), but not for naive cells. Specific group differences tested by ANOVA: naive cells, not significant; memory cells, *P* = .047, .010, for CD4^+^ and CD8^+^, respectively; *P < .05 by post hoc test. Abbreviations: ANOVA, analysis of variance; HIV, human immunodeficiency virus; HIV-2-HV, HIV-2 infected with high viral load (>1000 copies/mL); HIV-2-LV, HIV-2 infected with low viral load (<100 copies/mL); IQR, interquartile range.

When turnover rates were modeled to derive proliferation rate constants (*p*), we found that CD4^+^ naive cell turnover was relatively unaffected by disease status, having a turnover rate of about 0.5%/day in all groups, equivalent to a lifespan of about 150 days (Table 3). Although a trend toward accelerated turnover in CD8^+^ naive cells in HIV-2-HV and HIV-1 was seen, it was not significant. In memory cells, turnover showed a significant trend when analyzed using the same ranking as immune activation (controls <HIV-2-LV <HIV-1 <HIV-2-HV; Figure 3; *P* < .01 for CD4^+^ and CD8^+^ memory cells). The most marked effect was seen in the CD8^+^ compartment, where memory cell turnover was doubled in HIV-2-HV from 1.55% (controls) to 3.44%/day, while changes in memory turnover in HIV-2-LV were minimal compared to control subjects (Table 3; Figure 3). Not unexpectedly, disappearance kinetics (d*) did not differ between groups; they contribute to calculated values for proliferation, but, in short-term labeling studies such as this, contribute little as independent parameters, referring only to the small fraction of labeled cells [28].

#### TREC Content

**Figure 4.**
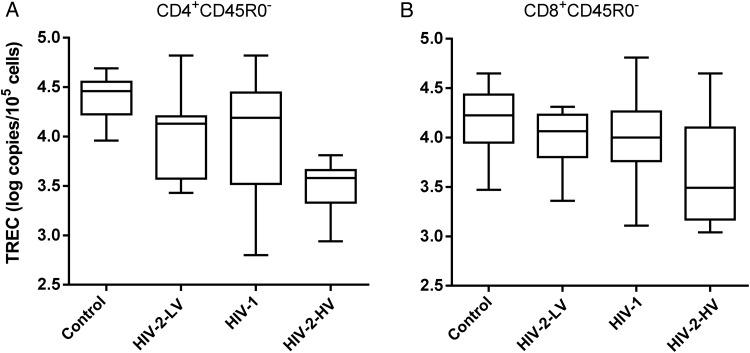
TREC content of naive CD4^+^ and CD8^+^ T-cells. TREC content shown as log copies per 10^5^ cells for (*A*) CD4^+^CD45R0^−^ and (*B*) CD8^+^CD45R0^−^ “naive” T-cells. Line, box, and whiskers represent median, IQR, and range, respectively. ANOVA (nonparametric): *P* = .004, *P* = .19 respectively; *significant intergroup difference by post hoc testing. Trend across groups was significant (*P* < .001) for CD4^+^ cells, with HIV-2-HV group having lowest TREC content values in naive cells, but not for CD8^+^ cells (*P* = .056). Abbreviations: ANOVA, analysis of variance; HIV, human immunodeficiency virus; HIV-2-HV, HIV-2 infected with high viral load (>1000 copies/mL); HIV-2-LV, HIV-2 infected with low viral load (<100 copies/mL); IQR, interquartile range; TREC, T-cell receptor excision circle.

TREC content showed the same hierarchy as described above in CD4^+^ cells, but reversed, with the lowest values in naive T-cells of HIV-2 high viral load subjects (Figure 4; Table 2). Age was an independent predictor of TREC content (*P* = .004), but, after controlling for age by multiple regression, the effect of infection status was still independently significant (*P* = .022). CD8^+^ cells appeared to show a similar hierarchy, but this was not significant (Figure 4).

### Predictors of T-cell Turnover in HIV-2 Infection

In order to understand the factors contributing to the known risk of HIV-related disease in the four subject groups (ie, none in controls, low-risk in HIV-2-LV [20], and high-risk in HIV-2-HV and HIV-1), we investigated relationships between parameters. For memory CD4^+^ T-cells, we found that, although proliferation rates were associated with viral load (Supplementary Figure 2*A*) and CD4 lymphopenia (Supplementary Figure 2*B*), the strongest association was with immune activation (HLA-DR/CD38 coexpression; Supplementary Figure 2*C*), although it should be noted that no markedly lymphopenic subjects were recruited. As expected, in vivo proliferation and Ki67 expression showed a significant correlation (*P* < .005; Supplementary Figure 2*D*), although not directly measuring the same physiological process.

For memory CD8^+^ T-cells, even stronger relationships were seen; immune activation strongly predicted in vivo turnover (Supplementary Figure 2*G*; *r* = .796, and *P* < .001) and Ki67 expression (0.696; *P* < .001). As for CD4^+^ T-cells, relationships with viral load and CD4 lymphopenia were weaker, but still significant (Supplementary Figure 2*E* and 2*F*). Interestingly, even in the control group there was a correlation between HLA-DR/CD38 coexpression and modeled proliferation rate (data not shown), suggesting that these methodologies are sufficiently sensitive to demonstrate subclinical inflammation or infection in clinically “healthy” controls.

## DISCUSSION

Control of HIV, with low or absent viremia, well-maintained CD4 counts, and absence of clinical disease, may be achieved in several ways. Elite controllers, for example, who represent about 1% of HIV-1 infected subjects, control viremia through strong CD8^+^ cytotoxic T lymphocyte (CTL) responses (with CD8^+^ T-cell activation); they have favorable HLA types for HIV-epitope presentation. A different pattern is seen in “posttreatment controllers,” who remain aviremic even after discontinuation of ART initiated during primary infection; their well being appears to result from low levels of immune activation rather than strong CTL responses [36]. Both patterns have wider implications as they inform pursuit of the goal of “functional cure,” control of viremia without ART. HIV-2 infection represents a further informative model, as subjects at very low and at high risk of disease progression can be differentiated according to viral load status [4]; indeed HIV-2-LV subjects in this cohort had a median untreated progression-free follow-up of approximately 12 years (Table 1). This dichotomy allows dissection of likely protective factors from those predisposing to pathology [1, 2]. Although a contribution from viral diversity cannot be excluded (for example, HIV-2 capsid proteins, such as p26, appear to favor progressive disease [37]), this study focuses on immunological factors.

First, our observations confirm the divergence between low and high viral load subjects with HIV-2 infection [2, 4]. We found clear differences between these groups in terms of immune activation, in vivo memory T-cell turnover, and Ki67 expression. Second, we found that the hierarchy of immune activation (controls <HIV-2-LV <HIV-1 <HIV-2-HV) predicted kinetic parameters, including Ki67 expression, in vivo turnover, and TREC content. Third, we found that although both naive and memory cells showed an activated phenotype in high viral load HIV-2-infected individuals, the effect on proliferation was more marked in the memory T-cell compartment. These 3 observations were more marked for CD8^+^ T-cells. Fourth, while in low viral load HIV-2, TREC were relatively well preserved, consistent with well-preserved telomeres in this group [38], we found that TREC content tended to be lower in HIV-1 and HIV-2 progressors; more markedly in CD4^+^ cells.

Our findings thus concur with the paradigm that immune activation is central to disease progression. Immune activation was the strongest predictor of T-cell turnover in both CD4^+^ and CD8^+^ populations (Supplementary Figure 2). This is consistent with the observation that, at similar clinical disease stages, HIV-1- and HIV-2-infected subjects demonstrate similar levels of immune activation [21, 39]. Our data add subset specificity to this paradigm; for example, naive CD8^+^ T-cells are even more activated by HIV-2 viremia than by HIV-1 (Figure 1*B*), consistent with poor outcomes at viral loads about 1 log_10_ lower in HIV-2 infection [9]. In parallel studies relating viral tropism to T-cell turnover, we also found that immune activation was the primary determinant of turnover rather than viral specificity or lymphopenia [25]. Consistent with this model is the extreme example of the SIV_sm_–infected sooty mangabey in which the absence of immune activation may explain how high-level viremia is tolerated without disease [40]. We interpret the association of CD4 lymphopenia with both CD4^+^ and CD8^+^ T-cell turnover (Supplementary Figure 2) as an indirect effect of immune activation rather than homeostasis, as CD4 lymphopenia affected both pools, whereas CD8 lymphopenia affected neither (data not shown). Drivers of immune activation include loss of gastrointestinal integrity and activation of toll-like receptors by viral RNA [18]. The final pathway for CD4 cell death may be caspase-1-mediated pyroptosis [41, 42].

We have previously observed the relative impunity of naive T-cells to the effects of viral infection on turnover in both HIV and acute Epstein–Barr virus infection [25, 43]. In HIV-2 high viral load patients, although we did not measure a change in turnover rate versus other groups, there still appears to be an effect on naive CD4^+^ cells, which have higher activation levels (Figure 1*A*) and lower TREC abundance (Figure 4). TREC content is determined both by thymic output and by replicative history [44]. We attribute the independent effect of age to reduced thymic output, but hypothesize that the HIV-2-HV effect relates to greater cumulative mitotic history in this group. Reduced TREC levels have also been demonstrated in Portuguese subjects with HIV-2 infection, albeit younger patients with undetectable viral loads [45]. Our data suggest that HIV-2 viremia accelerates TREC dilution. The relative absence of an effect of HIV-1 infection on TREC is consistent with other studies in early chronic HIV infection where thymic output appears to maintain TREC-high naive cell numbers [46]. Although all our subjects were in early chronic disease (in terms of CD4 count), later involvement of the naive T-cell pool may be a critical tipping point for progression, as naive cells are normally only very slowly replaced [26, 27].

The absence of differences in cellular markers of cell death or “exhaustion” (Annexin V, PD-1, 7AAD and CD95) may be the consequence of selecting a cohort with early disease (entry CD4 count > 500 cells/μL). Although previous studies have reported increased PD-1 expression in HIV-1 infection, this primarily affects virus-specific CD8^+^ [47] and antigen-specific CD4^+^ T-cells [48]. Other studies have found a less-marked effect of HIV-2 on PD-1-expression, consistent with our data [49]. The higher expression of Bcl-2 (which is antiapoptotic) in HIV-2-HV subjects may represent a protective mechanism operating at high CD4 counts, before cells are lost.

In vivo T-cell kinetics are technically difficult to estimate, but labeling approaches measuring stable isotope incorporation into DNA of dividing cells using either heavy water (deuterium oxide) or deuterium-labeled glucose have enabled direct noninvasive monitoring in humans [30]. Small changes may be difficult to detect, hence the major limitation of this study is its size; further larger studies, including studies investigating specific memory T-cell subpopulations and more precisely defined naive T-cells, would elucidate our observations. Our definition of “naive” cells may be adequate for CD4^+^ cells, but for CD45R0^−^CD8^+^ cell preparations would also have included some CD45RA-revertant “memory” cells [32]. Our decision not to investigate further T-cell subpopulations was based on logistic constraints; we limited our studies to subpopulations we could prepare in sufficient numbers by magnetic bead isolation (rather than flow cytometry), using limited blood sample volumes (a culturally sensitive issue), sorting only fresh cells (on the day of blood drawing), while promoting technology transfer by performing as much laboratory work on-site in The Gambia as possible. All samples were checked for purity by flow cytometry; sorted subsets that failed to meet a priori purity criteria were excluded. Minor contamination (naive T-cells in memory cells, or vice versa) was corrected for mathematically (Supplementary Information). Some control subjects may have had subclinical infection or low-grade inflammation, as illustrated by increased HLA-DR/CD38 coexpression and turnover in some control subjects, although they do represent a true picture of the background asymptomatic state in The Gambia and so represent valid controls. Investigation of the relationship between viremia, cell turnover, and the size of latent viral reservoirs would also be instructive.

The model of non-progression in HIV-2-LV subjects that emerges from this study is characterized by low, but not absent, levels of immune activation; indeed some CD8^+^ T-cell activation may be beneficial. HIV-2-LV subjects resemble HIV-1 elite controllers in that they have robust Gag-targeted polyfunctional T-cell responses [11, 13, 50], whose efficacy may be enhanced by greater constraints on CTL escape in HIV-2 infection [14]. The skewing of the cytokine profile of such cells away from perforin/cytotoxicity may explain the absence of immunopathology. In HIV-2, as in HIV-1, accelerated turnover appears to be a critical component of the pathological process leading to chronic cell depletion [41, 42]. Low levels of immune activation may protect cells from chronic depletion, as seen in posttreatment controllers [36]. Lessons from HIV-2 suggest that a “functional cure” response needs to balance sufficient targeted anti-HIV immune reactivity to critical epitopes with an absence of generalized aberrant immune activation, and specifically protection of the naive T-cell pool against exhaustion.

## Supplementary Data

Supplementary materials are available at *The Journal of Infectious Diseases* online (http://jid.oxfordjournals.org/). Supplementary materials consist of data provided by the author that are published to benefit the reader. The posted materials are not copyedited. The contents of all supplementary data are the sole responsibility of the authors. Questions or messages regarding errors should be addressed to the author.

Supplementary Data
