# Supplementary material for: Protection Versus Pathology in Aviremic and High Viral Load HIV-2 Infection—The Pivotal Role of Immune Activation and T-cell Kinetics
Source: J Infect Dis. 2014 May 5;210(5):752–61. doi: 10.1093/infdis/jiu165 (PMC4130319; doi:10.1093/infdis/jiu165)
Supplement: Supplementary Data [file supp_210_5_752__index.html]

Protection Versus Pathology in Aviremic and High Viral Load HIV-2 Infection—The Pivotal Role of Immune Activation and T-cell Kinetics — Supplementary Data 

# Protection Versus Pathology in Aviremic and High Viral Load HIV-2 Infection—The Pivotal Role of Immune Activation and T-cell Kinetics

## Supplementary Data

Supplementary Data

**Files in this Data Supplement:**

- Supplementary Data - Docx file
