# Supplementary material for: Protection Versus Pathology in Aviremic and High Viral Load HIV-2 Infection—The Pivotal Role of Immune Activation and T-cell Kinetics
Source: J Infect Dis. 2014 May 5;210(5):752–61. doi: 10.1093/infdis/jiu165 (PMC4130319; doi:10.1093/infdis/jiu165)
Supplement: Supplementary Data [file supp_jiu165_jiu165supp.docx]

**Supplementary Information**

**Protection versus pathology in aviremic and high viral load HIV-2 infection – the pivotal role of immune activation and T-cell kinetics**

Andrea Hegedus, Samuel Nyamweya, Yan Zhang, Sheila Govind, Richard Aspinall, Alla Mashanova, Vincent A.A. Jansen, Hilton Whittle, Assan Jaye, Katie L. Flanagan, Derek C. Macallan

**Supplementary methods**

**Immunophenotyping**

Three flow cytometry staining panels were used for immunophenotyping of freshly isolated whole blood as follows: Panel 1: CD4 APC-Cy7, CD8 PB, CD45RA APC, CD38 PE-Cy7, HLADR PerCP, Ki67 FITC, Bcl-2 PE; Panel 2: CD4 PE-Cy7, CD8 PB, CD45RA APC, PD-1 PE, 7AAD PerCP, Annexin V FITC; Panel 3: CD3 PE-Cy7, CD4 PerCP, CD8 PB, CD45RA APC, CD45RO PE, CD95 FITC. All antibodies were obtained from BD Biosciences except for Annexin V FITC, CD3 PE-Cy7 and CD8 Pacific Blue that were supplied by eBiosciences, UK. The surface antibodies (all apart from Ki67, Bcl-2 and Annexin V) were added to 50 μl heparinised blood as cocktails and incubated for 30 minutes in the dark at 5ºC and washed with FACS buffer. Panel 3 cells were then lysed with red blood cell lysing solution (10x dilution) (BD Biosciences), washed and then fixed with 1% formalin (FACS fix). For panel 1, cells were lysed, then permeabilized with Cytoperm/Cytowash (BD Biosciences), washed with BD Permwash (BD Biosciences), and incubated with 5 μl Ki67-FITC and 10 μl Bcl-2-PE for 30 minutes at 5ºC, washed with Permwash then FACS buffer and fixed with FACS Fix. For panel 2 cells were washed with 1:10 diluted Annexin buffer (provided with the Annexin V antibody) followed by 2.5μl of Annexin FITC. Plates were incubated in the dark at room temperature for 15 minutes, lysed as above, washed and fixed. One set of compensation samples was prepared for each panel using CD3 conjugated antibodies, and samples were treated the same as the test cells. All samples were either acquired immediately, or stored at 5ºC and acquired within 24 hours using a CyAn Advanced Digital Processing (ADP) High-Performance Flow Cytometer (DakoCytomation, Denmark).

Ex-vivo flowcytometry data were analysed using Flowjo software (Treestar, USA) and the graphical representations prepared using GraphPad Prism software (California, USA). The lymphocyte population was gated first, followed by CD3+ cells, which were then separated into CD4+ and CD8+ T-cells. These were then gated for CD45RA and/or CD45RO expression, followed by gating on the naïve and memory CD4 and CD8 populations and analysis for the markers of interest which were then compared between HIV-infected groups and healthy uninfected donors. An example of the gating procedure is shown in e-Figure 1.

**Cell sorting protocol**

*Magnetic cell sorting*

To obtain naïve (CD45RA+) and memory (CD45RO+) CD4+ and CD8+ T lymphocytes, cells were sorted magnetically using the Miltenyi Biotec system. The custom protocol used was based on the one described by Ghattas and colleagues in 2005(1). Naïve and memory cells were sorted with negative selection. We assumed that CD45RA- cells were CD45RO+ memory cells while CD45RO- cells were CD45RA+ naïve cells. This approach allowed us to sort only single CD45RA or CD45RO positive cells. From these fractions CD8+ and CD4+ cells were sorted positively as described below.

*CD45RA/CD45RO negative selection*

After discarding the supernatant, cells were resuspended in 3 ml T cell Kinetics Buffer (TK Buffer) made up of phosphate buffered saline (PBS) supplemented with 0.5% (w/v) bovine serum albumin (BSA) (VWR International, UK) and 2 mM EDTA. The solution was divided into aliquots of 1.8 ml and 1.2 ml in 5 ml Falcon tube labelled as RA and RO respectively. Tubes were topped up with TK Buffer and spun for 10 minutes at 1,800 rpm. After discarding the supernatant, 25 μl CD45RA MicroBeads and 20 μl CD45RO MicroBeads (both from Miltenyi Biotec GmbH, Germany) were added and tubes were incubated at +4^o^C for 15 minutes, mixing after 7 minutes. Samples were washed with TK Buffer for 10 minutes at 1,800 rpm then resuspended in 1 ml TK Buffer. For the separation steps, an OctoMACS Separator was used with appropriately labelled MS Columns and Pre-Separation Filters (all from Miltenyi Biotec GmbH, Germany). Cells were run through pre-wet columns. After rinsing columns with TK Buffer the cell solution was run through a second column to improve purity. Positively selected cells (CD45RA+ and CD45RO+) were flushed from the columns and kept refrigerated for later use. Negatively selected (CD45RA- and CD45RO-) cells were centrifuged for 10 minutes to prepare for CD8 positive sorting.

*CD8 positive selection*

After discarding the supernatant, 20-20 μl CD8 MicroBeads (Miltenyi Biotec GmbH, Germany) were added and cells incubated in the fridge as earlier. After washing, cells were run through two sequential columns, as described above. CD8- cells were stored at +4^o^C for CD4 sorting. CD8+ cells were then flushed from columns and spun for 10 minutes at 1,800 rpm. After resuspending in 1 ml TK Buffer, cells were run through a third column to increase purity and yield. Flushed cells were spun for 10 minutes at 1,800 rpm, resuspended in 1 ml TK Buffer and kept in the fridge after counting in 2 ml Sarstedt tubes labelled as CD8+CD45RA- and CD8+CD45RO- respectively.

*CD4 positive selection*

CD8- cells were centrifuged for 10 minutes at 1,800 rpm then labelled with 20-20 μl CD4 MicroBeads (Miltenyi Biotec GmbH, Germany) and run through 3 sets of columns as above. Cells were resuspended in 1 ml TK Buffer, counted and kept at +4^o^C in 2 ml Sarstedt tubes labelled as CD4+CD45RA- and CD4+CD45RO- respectively.

*Purity analysis of sorted cell populations with flow cytometry*

100 μl of PBMC and 40 μl of each cell populations were stained with 25 μl purity cocktail made up of: 5 μl anti-CD14-FITC, 5 μl anti-CD45RO-PE, 3 μl anti-CD4-PerCP, 3 μl anti-CD3-PE-Cy7, 2 μl anti-CD8-PB and 5 μl anti-CD45RA- (APC). (FITC, PE, PerCP and APC from BD Biosciences, USA; PE-Cy7 and PB from eBioscience Inc., USA) diluted in 6.5 μl FACS Buffer (PBS supplemented with 0.5% (w/v) BSA, 0.1% (w/v) EDTA and 0.1% (w/v) sodium azide (VWR International, UK)) per test. CD45RA+ cells mixed with CD45RO+ cells were used for unstained and compensation. Compensation tubes were added 5 μl FITC, 5 μl PE, 3 μl PerCP, 3 μl Pe-Cy7, 2 μl PB and 5 μl APC respectively. Samples were incubated at +4^o^C for 30 minute covered with kitchen foil to keep it in dark. Samples were washed in 2 ml of FACS buffer at 1,800 rpm for 10 minutes then fixed with 150 μl FACS Fix (1% (v/v) formalin in PBS). Acquisition was performed with CyAn ADP High-Performance Flow Cytometer (DakoCytomation, Denmark). Data were analysed with FlowJo Flow Cytometry Analysis Software (Tree Star Inc, USA).

**Estimation of deuterium enrichment in DNA**

DNA was extracted from sorted T-cell subpopulations and derivatized to the pentafluoro tri-acetate derivative by reaction with O-(2,3,4,5,6-Pentafluorobenzyl)hydroxylamine hydrochloride (PFBTA) (Sigma Aldrich). The derivative was analyzed for M+2 enrichment (*m/z* 437/435) in NCI mode by gas chromatography mass spectrometry (GC/MS) (2;3). Data were collected in triplicate after abundance matching with a standard curve of known enrichment.

Peak incorporation of deuterium into deoxyadenosine of DNA of labeled cells was taken as a surrogate measure of proliferation. Data were modeled to derive proliferation (*p*) and death rates (*d**) using the equation:

where, A = total deoxyadenosine, A* = labelled deoxyadenosine, p is proliferation rate (relative to all cells), d* is the disappearance rate (of labelled cells), and f(t) is a function to describe label availability. This model allows for disparity between proliferation rates, which relate to the whole population, and death rates, which relate only to labeled cells (4). Where there was minor contamination of memory cells in sorted naïve cells, or vice versa, a correction was performed based on the measured enrichment of the mixture and the known proportions of each cell-type from phenotypic analysis.

**Quantification of TREC**

Absolute quantification was performed as previously described (5). Briefly, DNA extracted from CD4^+^CD45R0^-^ and CD8^+^CD45R0^-^ cells was validated for quantity and integrity by amplification with albumin housekeeping primers before TREC determination with forward 5’-GGCAGAAAGAGGGCAGCCCTCTCCAAG-3’ and reverse 5’-GCCAGCTGCAGGGTTTAGG-3’ primers. All qPCR reactions were prepared robotically in triplicate using the QIAgility and performed on the Rotor-Gene Q (Qiagen GmbH). A 10µl total reaction mix comprising of 5µl of 2x QuantiTect SYBR (Qiagen GmbH), 0.5µM forward and reverse primers, genomic DNA 50ng was subjected to standard cycling conditions; hot start at 95^o^C for 15 min, and 40 cycles of 95^o^C denaturation for 15s, annealing for 30s at 61^o^C, followed by extension at 72^o^C for 30s. The specificity of the qPCR amplification was validated by melt-curve analysis using the Rotor-Gene Q software. Absolute quantification of viral copy number and TREC was determined by running a log linear 10^8^-10^1^standard curve using plasmid constructs of known copy number alongside unknown samples. The copy number of TREC was calculated by linear regression from the cycle threshold (Ct). Values are expressed as TREC/10^5^ CD4^+^CD45R0^-^ or TREC/10^5^ CD8^+^CD45R0^-^ cells.

**References for Supplementary Information**

(1) Ghattas H, Darboe BM, Wallace DL, Griffin GE, Prentice AM, Macallan DC. Measuring lymphocyte kinetics in tropical field settings. Trans R Soc Trop Med Hyg **2005**;99:675-85.

(2) Busch R, Neese RA, Awada M, Hayes GM, Hellerstein MK. Measurement of cell proliferation by heavy water labeling. Nat Protoc **2007**;2:3045-57.

(3) Macallan DC, Asquith B, Zhang Y, et al. Measurement of proliferation and disappearance of rapid turnover cell populations in human studies using deuterium-labeled glucose. Nat Protoc **2009**;4:1313-27.

(4) Asquith B, Debacq C, Macallan DC, Willems L, Bangham CR. Lymphocyte kinetics: the interpretation of labelling data. Trends Immunol **2002**;23:596-601.

(5) Mitchell WA, Lang PO, Aspinall R. Tracing thymic output in older individuals. Clin Exp Immunol **2010**;161:497-503.
